# Plasma Extracellular Vesicle Subtypes May be Useful as Potential Biomarkers of Immune Activation in People With HIV

**DOI:** 10.20411/pai.v6i1.384

**Published:** 2021-01-14

**Authors:** Wilfried Wenceslas Bazié, Julien Boucher, Julien Vitry, Benjamin Goyer, Jean Pierre Routy, Cécile Tremblay, Sylvie Trottier, Mohammad-Ali Jenabian, Patrick Provost, Michel Alary, Caroline Gilbert

**Affiliations:** 1 Centre de recherche du CHU de Québec-Université Laval, Québec, QC, Canada; 2 Département de microbiologie-infectiologie et d'immunologie, Faculté de médecine, Université Laval, Québec, QC, Canada; 3 Programme de recherche sur les maladies infectieuses, Centre Muraz, Institut National de Santé Publique, Bobo-Dioulasso, Burkina Faso; 4 Chronic Viral Illness Service and Division of Hematology, McGill University Health Centre, Montreal, QC, Canada; 5 Infectious Diseases and Immunity in Global Health Program, Research Institute, McGill University Health Centre, Montréal, QC, Canada; 6 Centre de recherche du Centre Hospitalier de l'Université de Montréal, Montréal, QC, Canada; 7 Département de microbiologie, infectiologie et immunologie, Faculté de médecine, Université de Montréal, Montréal, QC, Canada; 8 Département des sciences biologiques, Université de Québec à Montréal (UQAM), Montréal, QC, Canada; 9 Département de médecine sociale et préventive, Faculté de médecine, Université de Laval, Québec, C, Canada; 10 Institut national de santé publique du Québec, Québec, QC, Canada

**Keywords:** biomarkers, HIV-1, extracellular vesicles, immune activation, inflammation, CD4/CD8 ratio, microRNA, miR-92, miR-155, miR-223

## Abstract

**Background:**

Extracellular vesicles (EVs) are intercellular messengers with epigenetic potential since they can shuttle microRNA (miRNA). EVs and miRNA play a role in human immunodeficiency virus (HIV) infection immunopathogenesis. Chronic immune activation and systemic inflammation during HIV infection despite effective antiretroviral therapy (ART) are associated with non-acquired immunodeficiency syndrome (AIDS) comorbidities in people living with HIV (PLWH). Analysis of plasma EVs and their miRNA content may be useful as immune activation or inflammatory biomarkers in PLWH receiving ART. In this study, we hypothesized that the number, size, and miRNA of large and small EVs could reflect immune activation associated with an elevated CD8 T-cell count or a low CD4/CD8 ratio in PLWH.

**Methods:**

Plasma EVs subtype purified from PLWH and uninfected controls were sized using dynamic light scattering and quantified using flow cytometry and acetylcholine esterase (AChE) activity. Expression of mature miRNAs miR-92, miR-155, miR-223 was measured by quantitative reverse-transcriptase polymerase chain reaction in EVs and leucocytes.

**Results:**

HIV infection induces increased production of small EVs in plasma. EV subtypes were differentially enriched in miR-92, miR-155, and miR-223. Positive correlations between CD8 T-cell count and large EVs abundance and small EVs AChE activity were observed. CD4/CD8 ratio was negatively correlated with small EV AChE activity, and miRNA-155 level per small EV was negatively correlated with CD8 T-cell count.

**Conclusions:**

These findings suggest that quantifying large or small EVs and profiling miRNA content per EV might provide new functional biomarkers of immune activation and inflammation.

## INTRODUCTION

Since antiretroviral therapy (ART) does not significantly reduce the generalized immune activation and inflammation during human immunodeficiency virus (HIV) infection [1, 2], it is necessary to identify factors that underlie this persistent inflammation. Reliable biomarkers of treatment effectiveness and residual immune activation and inflammation will be essential to improve patient quality of life. Potential biomarkers begin to appear in the plasma during the early stages of infection. Membranous subcellular bodies called extracellular vesicles (EVs) are secreted during communication between dendritic cells, CD4 T cells, and other immune cells that play roles in HIV infection progression [3–9]. EVs can be recovered from body fluids such as blood, plasma, serum, urine, saliva, amniotic fluid, and breast milk [10]. Since their membrane, protein, and RNA composition reflect those of the cells or tissue of their origin, EVs may indicate the disease status [11–13]. Thus, they could become biomarkers for non-invasive investigations and clinical diagnostics and prognostics for pathogenesis, immune activation, inflammatory status, and perhaps ART resistance [14].

Based on current knowledge of EV biogenesis, there are 2 main categories: exosomes or small EVs and microvesicles or large EVs [15, 16]. Exosomes are intraluminal vesicles ranging from 30 to 100 nm in diameter that are formed by the inward budding of endosomal membranes during multivesicular endosome (MVE) maturation. Exosomes are secreted when MVEs fuse with the cell surface [16, 17]. Microvesicles range from 50 to 1,000 nm in diameter and are generated by the outward budding and fission of the plasma membrane and direct release into the extracellular space [16, 18].

Previous studies have shown that EVs play critical roles in virus pathogenesis by regulating cellular functions that may inhibit or enhance viral replication [19–24]. The biological activities attributed to EVs mainly depend on their cellular origin and specific bioactive molecule content such as proteins, lipids, DNA, mRNA, microRNA (miRNA), and other non-coding RNA [25, 27]. These component levels depend mainly on the functional states of cells that secrete EVs [19]. Indeed, biological fluid EVs might precisely reflect overall immune activation or inflammatory states.

Mounting evidence suggests that EVs and short, non-coding miRNA molecules are involved in HIV infection [21, 22, 28–30]. EVs exert regulatory effects on recipient cell physiological functions via shuttled miRNA [31–34], and contact with HIV alters miRNA expression in immune cells [35, 36]. Thus, miRNA could impact HIV infection pathophysiology in at least 2 ways. First, they could bind directly to HIV-1 RNA through base-pair complementarity to interfere with HIV genomic replication [37–39] or to mRNA to block viral protein translation [29, 38, 39]. Second, miRNA could also act indirectly by targeting host cell factors called “HIV dependency factors” [40–42] or immune cell pathways that suppress or activate viral replication [43, 44]. Among the miRNA molecules believed to play some role in HIV infection, we are interested in miR-155 and miR-223, which have been implicated in immune activation and inflammation, whereas miR-92 is involved in tumorigenesis and lymphoid differentiation.

A multifunctional miRNA, miR-155 is involved in innate and adaptive immune responses such as T helper cell differentiation, particularly Th17 and regulatory T cells in people infected with HIV-1 [45, 46]. It can regulate host dependency factors involved in the trafficking and nuclear importation of pre-integration complexes such as A Disintegrin And Metalloproteinase 10 (ADAM10), Transportin-3 (TNPO3), Nucleoporin Protein 153 (Nup153), and Lens Epithelium-Derived Growth Factor/protein75 (LEDGF/p75) during HIV-1 infection [47, 48]. To date, it is the only miRNA that can distinguish elite, long-term non-progressors from naive patients, and it is up-regulated in peripheral blood mononuclear cells (PBMCs) from naive HIV-1 infected patients in conjunction with pathogenesis [35]. It has also been shown to block expression of the E3 ubiquitin ligase TRIM32 by binding to the transcript's 3' untranslated region, which ultimately suppresses reactivation of latent HIV-1 [47, 49].

The microRNA, miR-223 is known to regulate the differentiation of several key players in the innate immune response (eg, neutrophils, monocytes, and granulocytes) and likely participates in the early stages of HIV infection and subsequent inflammation [50–52]. Its expression is positively correlated with the susceptibility of T cells, monocytes, and macrophages to infection, and this is more substantial in resting CD4 T cells (in which HIV replication is silenced) than in activated CD4 T cells. Inhibition of miR-223 or in combination with other miRNAs has been shown to enhance HIV-1 replication in resting CD4 T cells by indirectly affecting cyclin T1 levels [39, 53, 54].

The miR-92 members of the miR-17-92 cluster are distinguished as onco-miRs because of their importance in cell transformation and tumorigenesis [55]. The miR-17-92 cluster has emerged as a central integrator of gene expression events that govern T helper cell differentiation pathways. T-cell overexpression of miR-17-92 promotes proliferation and decreases the apoptosis rate, leading to lymphoproliferative disease and autoimmunity [56]. In particular, miR-92a promotes human T follicular helper cell differentiation by targeting Krüppel-like factor 2 and PTEN [57].

In silico approaches to identifying intracellular binding sites of miRNAs that directly target HIV-1 RNA have shown that miR-155 binds to *vif*, miR-92 to the *pol* region, and miR-223 to the *nef* region of HIV thereby contributing to repressing HIV-1 replication [37–39, 53]. Experiments have shown that miR-92 can suppress HIV-1 replication in transfected cells by 40% [38]. How miRNA contents in various EV subsets are altered under different physiological and pathological conditions must be understood both conceptually and quantitatively before their usefulness as biomarkers can be proclaimed.

Before being encapsulated in EVs for delivery to other cells, miRNAs are generated in the cell within which they will also affect post-transcriptional regulation of gene expression. PBMCs are among the major circulating targets of HIV infection, and their miRNA content profile during HIV infection has been described by several studies [29, 30]. Polymorphonuclear cells (PMNs) are a subset of immune cells and the most abundant leukocytes in human blood. They are key components of the early immune innate response to HIV infection, contribute to viremic control, and activate and modulate the quality of the adaptive immune response [58, 59]. PMNs also contribute to the chronic immune activation observed during HIV-1 infection, which is associated with the development of non-HIV/AIDS-related inflammatory conditions, even in those with well-controlled viremia under highly active ART (HAART) [59–62]. Gomez *et al* showed that activated neutrophils exacerbate vascular inflammation by releasing pro-inflammatory microvesicles with increased miRNA content [63]. These vesicles contain several miRNAs including regulators of inflammation (eg, miR-9, miR-150, miR-155, miR-186, miR-223, and miR-23a) with potential effects on the pathophysiological processes underpinning vascular inflammation, atherogenesis, and intestinal inflammation [63–65]. Based on these roles, PMNs also participate in EV production [66, 67] similar to other eukaryotic cells found in plasma. It is essential to understand miRNA expression in cells (PBMCs and PMNs) and the extracellular environment (EVs) during HIV infection because the value of miRNA as a biomarker could depend on where it is detected.

Despite the great interest in EVs in various pathological conditions, their isolation and nomenclature have yet to be standardized [68]. While many studies focus on exosomes, EVs are highly diverse, and it is crucial to consider differences in the secretion mechanisms and contents of their subtypes from one disease stage to the next [69]. According to our hypothesis, depending on the biogenesis mode, specific EV types contain miRNA that leads to increased CD8 T-lymphocyte counts that characterize residual immune activation in ART-treated patients. This study aimed to characterize EV subpopulations and describe miRNA content in EVs, and lymphoid, and myeloid cells. The expression levels of miR-92, miR-155, and miR-223 in large and small plasma EVs in HIV-infected and uninfected patients were investigated along with relationships between EV type, miRNA content, and clinical parameters, and between cellular and vesicular miRNA.

## MATERIALS AND METHODS

### Population Study

PLWH treated with ART with plasma viral load ≤ 50 copies/mL (n = 11) and ART-naive PLWH (n = 6) were recruited at “Unité Hospitalière de Recherche, d'Enseignement et de Soins du SIDA/VIH/hépatite (UHRESS) CHU de Québec Université Laval, CHUL.” Uninfected control participants (n = 8) were also included. The characteristics of the participating patients and healthy volunteers are presented in Table 1. Comparing immunological characteristics of PLWH, we observed that patients successfully treated with ART have lower nadir CD4 count means (± standard deviation) 259 ± 106 vs 406 ± 128 cells/µL, *P* = 0.0192) and longer duration of infection (18.4 ± 8.9 years vs 5 ± 4.9 years, *P* = 0.0031) than ART-naive patients. No significant differences were observed for CD4 T-cell count, CD8 T-cell count, or CD4/CD8 ratio. The difference in nadir CD4 count is attributable to infection duration because ART-naive patients were newly or early diagnosed (3/6), and other patients were considered long-term non-progressors (3/6).

**Table 1:** Characteristics of participants enrolled in the study

ID	Group	Age (years)	Sex	Duration of HIV infection (years)	Time on ART (years)	CD4 nadir (cells/μL)	HIV-1 RNA (copies/μL)	CD4 (cells/μL)	CD8 (cells/μL)	CD4/CD8 ratio	Current ART regimen
1	ART	23	Male	3.3	0.5	493	≤ 50	493	1763	0.28	ABC, 3TC, RPV
2	ART	60	Male	27.9	22.1	187	≤ 50	481	635	0.76	TDF, FTC, EFV, RAL, MVC
3	ART	60	Male	29.4	17.0	270	≤ 50	582	1253	0.45	TDF, FTC, NVP
4	Control	49	Male	-	-	-	-	-	-	-	-
5	Control	39	Male	-	-	-	-	-	-	-	-
6	Naive	29	Male	8.4		341	11234	395	597	0.65	
7	Control	33	Male	-	-	-	-	-	-	-	-
8	ART	42	Male	11.5	0.6	271	≤ 50	594	685	0.87	ABC, 3TC, RPV
9	Control	32	Female	-	-	-	-	-	-	-	-
10	Naive	48	Female	10.1	-	314	5608	470	1070	0.44	-
11	Control	56	Female	-	-	-	-	-	-	-	-
12	ART	62	Male	9.5	8.5	277	≤ 50	610	570	1.07	TDF, FTC, EFV
13	Control	46	Female	-	-	-	-	-	-	-	-
14	Naive	55	Male	0.2	-		266 413	969	1352	0.73	-
15	Control	45	Female	-	-	-	-	-	-	-	-
16	ART	60	Male	12.7	10.5	451	≤ 50	684	1077	0.64	ABC, 3TC, NVP
17	Naive	22	Male	0.9	-	465	3751	1433	3119	0.46	-
18	Naive	48	Male	10.4	-	564	60	714	774	0.91	-
19	ART	40	Male	13.3	13.3	138	≤ 50	910	480	1.37	ABC, 3TC, LPV, RTV
20	ART	58	Male	20.8	17.5	256	≤ 50	393	541	0.73	AZT, 3TC, FPV, RTV
21	Naive	58	Male	1.3	-	345	192300	432	902	0.48	-
22	ART	41	Male	20.4	16.2	126	≤ 50	441	676	0.65	TDF, FTC, LPV, RTV
23	ART	60	Male	25.0	22.4	76	64	491	757	0.65	TDF, FTC, EFV, RTV, ATV
24	Control	58	Female	-	-	-	-	-	-	-	-
25	ART	60	Male	28.8	13.5	304	≤ 50	517	535	0.97	TDF, FTC, RAL

**Abbreviations:** 3TC, lamivudine; ABC, abacavir; ART, antiretroviral therapy; ATV, atazanavir; AZT, zidovudine; DRV, darunavir; EFV, efavirenz; EVG, elvitegravir; FPV, fosamprenavir; FTC, emtricitabine; LPV, lopinavir; MVC, maraviroc; RAL, raltegravir; RTV, ritonavir; TDF, tenofovir disoproxil fumarate.

This study received approval from the ethics review boards of Centre de recherche du CHU de Québec, Québec. All participants were anonymous volunteers and provided written, informed consent to participate in the study.

### Cell Purification

Peripheral blood from venipuncture was collected in EDTA-containing tubes at the CHU de Québec, QC, Canada, in 2013. PMNs and PBMCs were isolated from fresh blood samples. Cells were purified as described previously [70, 71]. Cell pellets (20 million PMNs and 5 million PBMCs) were diluted in TRIzol for miRNA analysis.

### EV Purification

Blood obtained by venipuncture with citrate as anticoagulant was centrifuged for 10 minutes at 400*g* at room temperature to obtain plasma. Plasma was centrifuged for 10 minutes at 3,000*g* to obtain platelet-free plasma and stored at-80°C. Large EVs were separated from thawed plasma by centrifuging 250 µL in sterile Eppendorf tubes for 30 minutes at 17,000*g* at room temperature. The resulting supernatant was mixed with 63 µL of ExoQuick™ (SBI via Cedarlane, Burlington, ON, Canada) in an Eppendorf tube and held at room temperature for 30 minutes. The centrifugal pellet was mixed with 250 µL of micro-filtered (0.22-µm pore size membrane) 1X phosphate-buffered saline (PBS; WISENT Bioproducts, Saint-Jean-Baptiste, QC, Canada), and the suspension was centrifuged for 30 minutes at 17,000*g*. The supernatant was discarded, and the washed large EV pellet was re-suspended in 250 µL of PBS and kept at 4°C. Small EVs were obtained from the ExoQuick suspension by centrifuging for 30 minutes at 1,500*g*, discarding the supernatant, re-suspending the pellet in 250 µL of PBS, and centrifuging at 1,500*g* for 5 minutes. The washed small EV pellet was re-suspended in 250 µL of PBS by vortex mixing and kept at 4°C.

### EV Size Measurement

EV size was determined by hydrodynamic radius measurement using a Zetasizer Nano S (Mal-vern Instruments, Malvern, United Kingdom), which uses dynamic light scattering to estimate the diameter of particles in the 0.3 nm to 10 µm range. This technique is based on the fluctuation of light scattering intensity due to Brownian motion, characterized by a diffusion constant [72]. The size distribution was obtained from the distribution of diffusion constants using the Einstein-Stokes equation [72]. The size was measured in duplicate using 100 µL of EV suspension.

### Acetylcholinesterase Activity Assay

Acetylcholinesterase (AChE) activity was measured following a procedure described previously [13]. Briefly, EV samples (100 µL) were mixed with 1.25mM acetylthiocholine in PBS at pH 8 plus 0.1mM 5,5-dithio-bis (2-nitrobenzoic acid) in PBS at pH 7. The resulting suspension (200 µL) was held at room temperature and then warmed to 37°C for 10 minutes for optical density reading. Absorption at 450 nm was monitored for 10 minutes using a plate reader spectrophotometer (Molecular Devices with SOFTmax^®^ Pro version 5, San Jose, CA, USA).

### miRNA Quantification

Thawed plasma (250 µL) was treated with proteinase K (1.25 mg/mL, Ambion™, Thermo Fisher Scientific, Waltham, MA, USA) for 10 minutes at 37°C, and EVs were recovered as described above using the method and kit described previously [13]. EV suspensions (100 µL) were diluted 3:1 in TRIzol LS (Ambion, Life Technologies, Carlsbad, CA, USA) and held at-80°C. Total RNA was extracted from EVs, PBMCs, and PMNs; mixed with 10 µL of diethylpyrocarbonate water, and quantified (1 µL) using a BioDrop-μLITE kit (Isogen Life Science, Utrecht, Netherlands).

About 100 ng of RNA was treated with RNase-free DNase I (Ambion™ Life Technologies) and then reverse transcribed using a HiFlex miScript RT II kit according to the manufacturer's instructions (Qiagen, Hilden, Germany). Mature miR-92 (#MS00006594), miR-155 (#MS00031486), and miR-223 (#MS00003871) were detected by quantitative reverse-transcriptase polymerase chain reaction (qRT-PCR) using a miScript primer assay kit and miScript SYBR Green PCR kit (Qiagen). Amplification of mature miRNA as cDNA was performed in a CFX Connect real-time PCR Detection System (BIO-RAD, Hercules, CA, USA) using 40 cycles of 94°C for 15 seconds, 55°C for 30 seconds, and 72°C for 30 seconds. Reaction specificity was confirmed using the melt curve procedure (65–95°C, 0.5°C per 5 seconds) at the end of the amplification protocol according to the manufacturer's instructions. A standard curve was used for absolute miRNA quantification. Using synthetic hsa-miR-92, hsa-miR-155, and hsa-miR-223, an improved standard curve was obtained for miRNA concentrations ranging from 10^3^ to 10^8^ molecules.

### EV Flow Cytometry Analysis

A method described previously [73] for microparticle analyses was used for EV analysis by flow cytometry in a FACS Canto II Special Order Research Product cytofluorometer equipped with forward scatter coupled to a photomultiplier tube (FSC-PMT) with the “small particles option” (BD Biosciences, Franklin Lakes, NJ, USA). Purified EVs were stained with the lipophilic fluores-cent carbocyanine dye DiD (DiIC_18_ (5) solid: 1,1'-dioctadecyl-3,3,3',3'-tetramethylindodicarbocyanine 4chlorobenzenesulfonate salt, Invitrogen™, Carlsbad, CA) and FITC-labeled antibodies directed against surface markers: anti-CD45, anti-CD4, and anti-CD8a (respectively products 11-9459-42, 110048-42, and 11-0089-42 from eBioscience^TM^, San Diego, CA). DiD was used at 5 µL/mL of EV sample with 0.02% Pluronic F-127 (Invitrogen). After 20 minutes at 37°C, 1 portion was used to count total EVs, and the other was divided into 3 aliquots of 10µL that were each mixed with antibody and held for 15 minutes at room temperature. Next, 100 µL of 4% paraformaldehyde was added, followed by 300 µL of filtered PBS 20 minutes later and counting by flow cytometry. The gating strategy for EV identification by high-resolution flow cytometry is shown in Supplementary Figures 1 A-D.

### Procedure for Calculating miRNA Copy Number Per Vesicle

Large EVs were separated from 250 µL of plasma, then mixed with 63 µL of ExoQuick. Total RNA was extracted from 100 µL of the small EVs and large EV fractions. Small EVs yielded 223 ng of RNA (from the 1.25X dilution due to ExoQuick), and large EVs yielded 260.6 ng (from 100 µL), which indicates that the plasma contained 2.8 ng/µL associated with small EVs and 2.6 ng/µL associated with large EVs. Based on RT-qPCR, miRNAs are expressed in copies/ng of RNA, and EV counts (obtained by cytofluorometry) are expressed per µL of plasma. Multiplying the miRNA copies/ng of RNA by the RNA per µL of plasma gives miRNA copies/µL. Dividing this result by EVs count per µL yields miRNA copies/EVs in 1 µL of plasma. By multiplying the result by a factor of 1000, we obtain the unit number of miRNA copies per vesicle in 1 ml of plasma.

### Statistical Analysis

Data are presented as box and whisker plots. The lines inside the boxes and the upper and lower limits of the boxes indicate the median, 75th, and 25th percentiles, respectively. The upper and lower horizontal bars indicate the maximum and minimum values, respectively. Non-parametric tests were used for comparisons. Wilcoxon matched-pairs signed-rank tests were used to compare individual paired data, and Kruskal-Wallis tests with Dunn's multiple comparisons were used to compare group mean rankings. All correlation coefficients were calculated using Spearman rank correlation, and *P* values are 2-tailed. Statistical analyses were performed using GraphPad Prism 8 (GraphPad Inc, San Diego, CA, USA). A *P* value ≤ of 0.05 was deemed statistically significant.

## RESULTS

### Characterization of Both Purified Plasma EV Subsets

**Figure 1.**
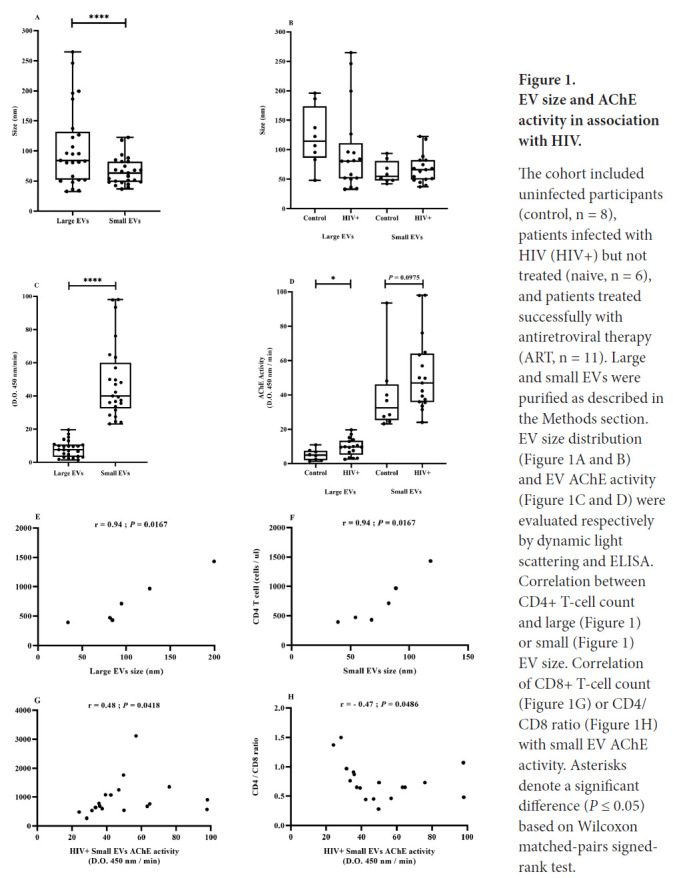
EV size and AChE activity in association with HIV. The cohort included uninfected participants (control, n = 8), patients infected with HIV (HIV+) but not treated (naive, n = 6), and patients treated successfully with antiretroviral therapy (ART, n = 11). Large and small EVs were purified as described in the Methods section. EV size distribution (Figure 1A and B) and EV AChE activity (Figure 1C and D) were evaluated respectively by dynamic light scattering and ELISA. Correlation between CD4+ T-cell count and large (Figure 1) or small (Figure 1) EV size. Correlation of CD8+ T-cell count (Figure 1G) or CD4/CD8 ratio (Figure 1H) with small EV AChE activity. Asterisks denote a significant difference (*P* ≤ 0.05) based on Wilcoxon matched-pairs signed-rank test.

Because of the previously noted heterogeneity of EV populations [13], EVs were separated into “large” and “small” subsets to facilitate their characterization. Large EVs were pelleted from plasma after 30 minutes centrifugation at 17,000*g.* The small EV fraction was co-precipitated with ExoQuick™ from the 17,000*g* centrifugation supernatant. The large EV fraction contained a wide range of sizes overlapping with the small size range (Figure 1A). A paired t-test showed that the size difference between large and small EVs was significant (*P* < 0.001). However, EV size did not differ significantly between control participants and PLWH (Figure 1B).

AChE activity, a generic marker of EVs of late endosome origin [74], also differentiated EV subsets. The AChE activity range was significantly greater in small EVs and did not overlap with the relatively limited range characterizing large EVs (Figure 1C). The AChE activity of large and small EVs gave means (SEM) of 8.2 (0.99) and 48.18 (4.51), respectively. The difference in AChE activity between healthy participants and HIV-infected patients was only statistically significant for the large EV fraction (Figure 1D).

The biomarker potentials of EV size and AChE activity in HIV infection were evaluated by correlation with clinical parameters. In ART-naive patients, large and small EV size was strongly and positively correlated with CD4 T-cell count (Figure 1E and F). Considering all HIV-infected participants, small EV AChE activity was positively correlated with CD8 T-cell count (Figure 1G) and inversely correlated with CD4/CD8 ratio (Figure 1H).

These correlations corroborate earlier observations [13] and reinforce the biomarker potential of plasma EVs in participants with HIV. They draw attention to the existence of EV subsets based on size and AChE activity and show that the method is useful for distinguishing both characteristics. However, AChE activity is associated with EV relative abundance and is not as good a qualitative measurement as accurate EV count and quantitative determination of cell origin.

### HIV-1 Infection Increases Small EV Release in Plasma

**Figure 2.**
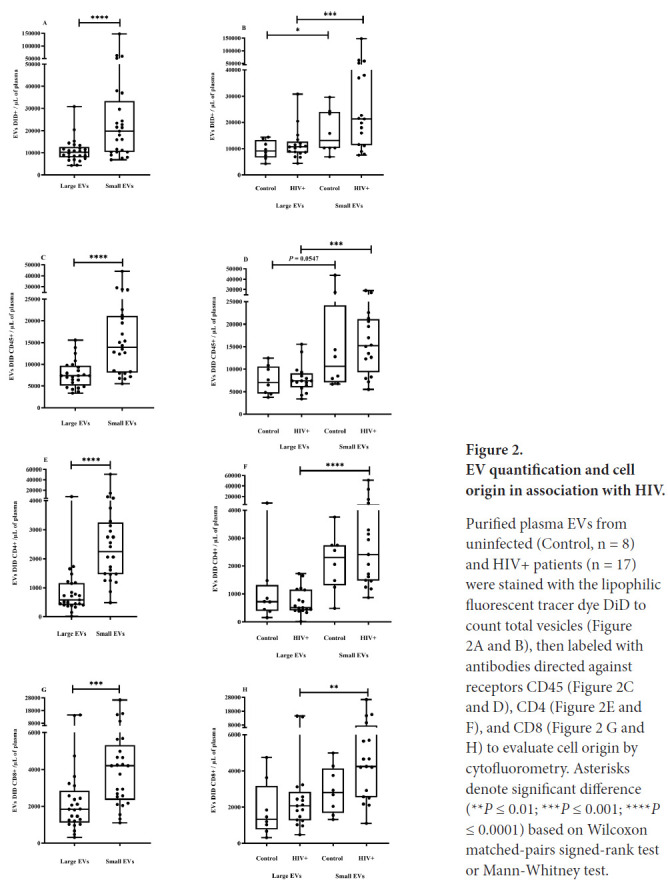
EV quantification and cell origin in association with HIV. Purified plasma EVs fromuninfected (Control, n = 8) and HIV+ patients (n = 17) were stained with the lipophilic fluorescent tracer dye DiD to count total vesicles (Figure 2A and B), then labeled with antibodies directed against receptors CD45 (Figure 2C and D), CD4 (Figure 2E and F), and CD8 (Figure 2 G and H) to evaluate cell origin by cytofluorometry. Asterisks denote significant difference (**P ≤ 0.01; ***P ≤ 0.001; ****P ≤ 0.0001) based on Wilcoxon matched-pairs signed-rank test or Mann-Whitney test.

During HIV-1 infection, several effector components of host innate and adaptive immune systems are activated to prevent the infection from gaining a foothold [75, 76]. To determine which cells shed most of the small and large EVs purified, and what impact HIV infection had on their production, EVs were counted using the lipophilic fluorescent tracer dye DiD for EV phospholipid bilayer membrane staining and antibodies directed against EV surface markers. An antibody against CD45 was used for EV leukocyte origin, and antibodies against CD4 and CD8 were used for cells bearing these receptors, particularly CD4 and CD8 T cells. We observed that the plasma concentration of small EVs was significantly higher, and this difference was observed in PLWH plasma despite their CD45+, CD4+, and CD8+ origins (Figure 2A-H).

**Figure 3.**
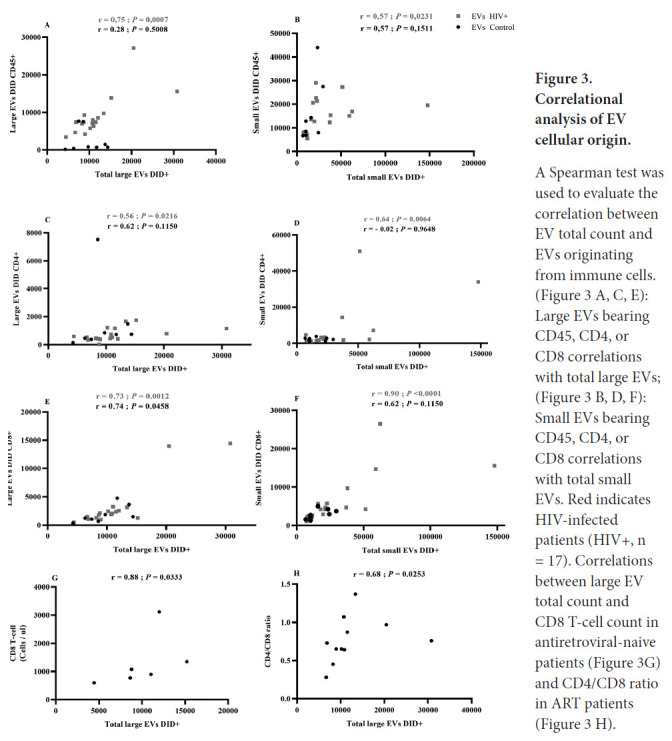
Correlational analysis of EV cellular origin. A Spearman test was used to evaluate the correlation between EV total count and EVs originating from immune cells. (Figure 3 A, C, E):Large EVs bearing CD45, CD4, or CD8 correlations with total large EVs; (Figure 3 B, D, F): Small EVs bearing CD45, CD4, or CD8 correlations with total small EVs. Red indicates HIV-infected patients (HIV+, n = 17). Correlations between large EV total count and CD8 T-cell count in antiretroviral-naive patients (Figure 3G) and CD4/CD8 ratio in ART patients (Figure 3 H).

We also measured correlations between receptor-bearing EV counts and total EV counts in the large and small vesicle categories (Figure 3). In PLWH, counts of large and small EVs bearing CD45, CD4, and CD8 were positively correlated with the total counts of large and small EVs (Figure 3 A–F). Excluding large EVs bearing CD8, these correlations were not observed in healthy participants (Figure 3A–F).

The count of cells bearing the CD8 marker was correlated with total large EV counts in ART-naive patients (Figure 3G), while the CD4/CD8 ratio had a weaker but still significant correlation with total large EV counts in the ART-treated cohort (Figure 3H). These results suggest that HIV infection induces increased production of small EVs in plasma, while associated correlations in PLWH denote more active functional participation of immune cells.

### Small and Large EV Subsets are Differentially Enriched with miRNA

**Figure 4.**
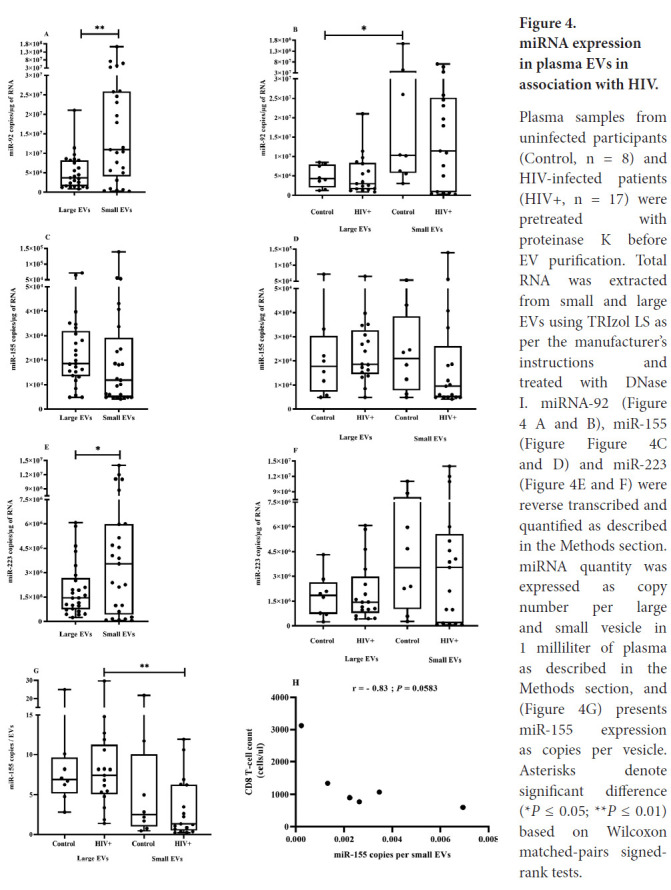
miRNA expression in plasma EVs in association with HIV. Plasma samples from uninfected participants (Control, n = 8) and HIV-infected patients (HIV+, n = 17) were pretreated with proteinase K before EV purification. Total RNA was extracted from small and large EVs using TRIzol LS as per the manufacturer's instructions and treated with DNase I. miRNA-92 (Figure 4 A and B), miR-155 (Figure 4C and D) and miR-223 (Figure 4E and F) were reverse transcribed and quantified as described in the Methods section. miRNA quantity was expressed as copy number per large and small vesicle in 1 milliliter of plasma as described in the Methods section, and (Figure 4G) presents miR-155 expression as copies per vesicle. Asterisks denote significant difference (*P ≤ 0.05; **P ≤ 0.01) based on Wilcoxon matched-pairs signed-rank tests.

Since miR-92, miR-155, and miR-223 were previously found to be more abundant in EVs from ART-naive PLWH, and miR-155 and miR-233 quantitatively correlated with EV abundance and size [13], we investigated the possibility that small and large EVs contain different amounts of these miRNA species. For this purpose, platelet-free plasma was treated with proteinase K before EV purification to eliminate plasma of free RNA bound to lipoproteins or vesicle surfaces [77]. Concerning EV miRNA content, we found that miR-92 and miR-223 were significantly more abundant in small EVs, independently of HIV status (Figure 4A, C, and E). HIV infection had no significant effect on miR-155 and miR-223 quantity in the EV subset, although the previously observed trend was preserved (Figure 4B, D, and F). The expected significant correlation between EV-borne miRNA and viral load, CD4 or CD8 T-cell count, or CD4/CD8 ratio was not found. Besides EV miRNA expression as copies per RNA microgram, we also considered the new measurement of miRNA copies per EV. We observed that miR-155 copies were more numerous in large vesicles than in small ones, and this difference was only significant in EVs from PLWH (Figure 4G and Supplementary Figure 2A and B). To evaluate the possibility of vesicular miRNA providing a new biomarker of HIV disease, we examined the relationship between EV miRNA expression level per vesicle and clinical parameters. In ART-naive PLWH, miR-155 copies per small vesicles were inversely correlated with CD8 T-cell count, although the result was not significant (Figure 4H, *P* = 0.0583). Copies of miR-223 per large vesicle were strongly and inversely correlated with viral load (Supplementary Figure 2C). These results suggested that some miRNAs are preferentially associated with a subset of EVs, and expression level per vesicle needs to be considered in addition to quantifying the miRNA content of EV fractions.

### Differential Expression of miRNA in PBMCs and PMNs

**Figure 5.**
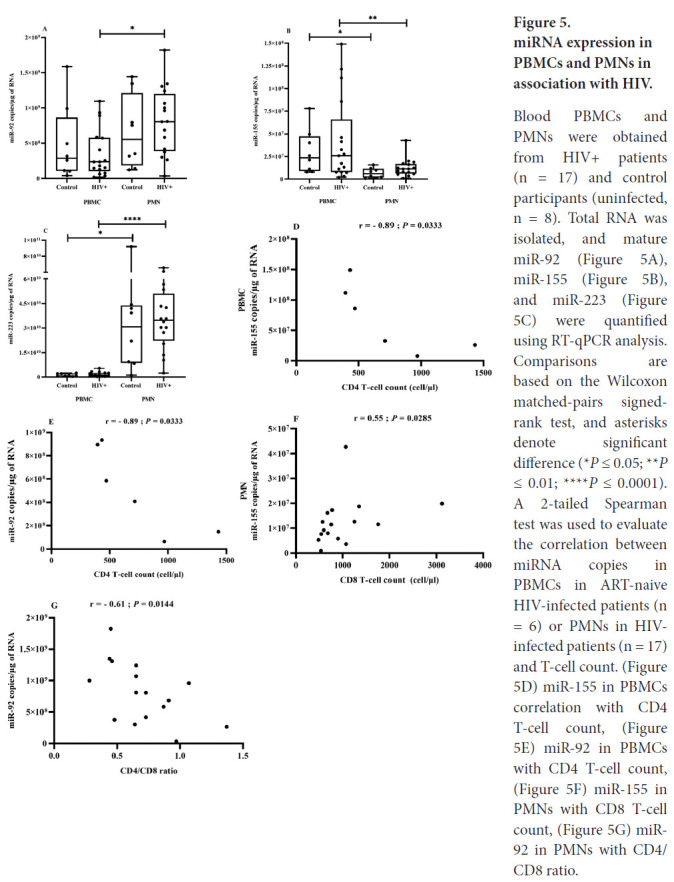
miRNA expression in PBMCs and PMNs in association with HIV. Blood PBMCs and PMNs were obtained from HIV+ patients (n = 17) and control participants (uninfected, n = 8). Total RNA was isolated, and mature miR-92 (Figure 5A), miR-155 (Figure 5B), and miR-223 (Figure 5C) were quantified using RT-qPCR analysis. Comparisons are based on the Wilcoxon matched-pairs signed-rank test, and asterisks denote significant difference (*P ≤ 0.05; **P ≤ 0.01; ****P ≤ 0.0001). A 2-tailed Spearman test was used to evaluate the correlation between miRNA copies in PBMCs in ART-naive HIV-infected patients (n = 6) or PMNs in HIV-infected patients (n = 17) and T-cell count. (Figure 5D) miR-155 in PBMCs correlation with CD4 T-cell count, (Figure 5E) miR-92 in PBMCs with CD4 T-cell count, (Figure 5F) miR-155 in PMNs with CD8 T-cell count, (Figure 5G) miR-92 in PMNs with CD4/CD8 ratio.

Despite numerous studies focused on miRNA profile changes in HIV-infected PMBCs, CD4, and CD8 T cells, PMNs have been largely neglected. We found that miR-223 and miR-92 were significantly more abundant in PMNs and miR-155 in PBMCs, but there were no significant differences between participants with HIV and controls (Figure 5A, B, and C). However, all 3 miRNA species tended to be more abundant in PBMCs from the ART-naive group.

Next, we correlated PBMC miRNA to well-known virologic and immunological markers of HIV (CD4 and CD8 T-cell counts and CD4/CD8 ratio). In ART-naive participants, miR-155 (Figure 5D) and miR-92 (Figure 5E) levels were strongly and inversely correlated with CD4 T-cell count.

No correlation was found in ART patients. These results show and confirm the role of PBMCs in HIV pathology and the relevance of miRNAs as biomarkers of HIV infection.

The tendency towards increased miRNA expression in PMNs of HIV-infected patients is shown in (Figure 5A-C). No significant difference was seen between ART-naive and ART-treated patients. Spearman correlational analysis showed that increasing miR-155 in PMNs was correlated with CD8 T-cell count (Figure 5F) and increasing miR-92 was inversely correlated with CD4/CD8 ratio (Figure 5G) in association with HIV infection. The value of miRNAs as biomarkers could depend on where they are detected, and their levels in PMNs could be a new functional biomarker of inflammation.

## DISCUSSION

Since the discoveries of EVs and miRNAs, evidence implicating them in human disease has mounted along with the expectation of their therapeutic and diagnostic use. In the case of miRNA, the roles of the various intracellular and extracellular forms must be elucidated before any molecule could be given serious consideration as a biomarker of HIV disease state, and the same is true for EV subsets.

Our team has shown previously that the size and AChE activity of EVs differ between PLWH and healthy participants and that these characteristics are correlated with T-cell counts and CD4/CD8 ratio [13]. To identify which EV subset is most strongly associated with these clinical parameters, we began by defining large and small size categories. In contrast with previous reports [78, 79], we found no significant difference in EV size category between uninfected participants and ART-treated or ART-naive PLWH. However, the correlation between exosome abundance (based on AChE assay) and CD8 T-cell count or CD4/CD8 ratio was corroborated in this study. AChE activity is more significant in small EVs than in large EVs and therefore has been described as a characteristic of EVs of late endosome origin (ie, exosomes) [80]. Further investigation is needed to confirm that small EVs are a reliable clinical proxy for exosomes and understand why exosomes are more abundant in HIV+ patients. Based on our results, the absence of correlation with viral load could mean that EV abundance is a marker of immune activation rather than viral replication.

Unlike AChE assays that only provide a qualitative estimation of EV relative abundance, flow cytometry provides absolute counts and quantitative detection based on fluorochrome-coupled antibodies directed against specific markers. Our results show that small EVs are more abundant than large EVs in plasma, independently of surface markers. This difference was only significant in HIV-infected patients regardless of staining (CD45, CD8, and CD4). Several studies have shown that HIV infection induces exosome release by immune cells [8, 9, 81, 82], and this could explain why CD45+, CD8+, or CD4+ EVs correlated with EV number in PLWH. Counts of EV subsets were correlated with CD8 T-cell count and CD4/CD8 ratio, and both parameters are used for clinical HIV infection management. Indeed, others have speculated that any increase in the number of circulating EVs in HIV-infected patients comes from a broad population of persistently activated immune cells [83, 84]. These results suggest that EV number could be a useful bio-marker for monitoring inflammation, immune activation, and immunosenescence during HIV infection and persists despite effective ART suppression of viral replication [85–87].

Mounting evidence suggests that specific miRNAs might be clinical biomarkers of many types of diseases including HIV infection [29]. The miRNA contents of EVs are best characterized in the case of exosomes [88, 89]. Changes in the miRNA profile of HIV-infected cells are well known, and the associated EVs should reflect them. Our results show that large EVs are enriched preferentially with miR-155. We found that miR-223 is more abundant in small EVs than in large EVs. A mechanism involving RNA binding protein Y-box 1 appearing to selectively load exosomes with miRNA-223 has been previously described [89]. HIV infection might create a network in which EVs are enriched with selected miRNAs for delivery to neighboring cells. It has been estimated that a threshold concentration of at least 100 copies of miRNA must be reached to downregulate mRNA expression [90]. Our results indicate that the number of copies per vesicle is low for miR-155 compared to miR-223 and miR-92. As observed by Chevillet *et al* [88], this low number of miRNA copies per vesicle implied that most EVs would not contain any copies of miRNAs. These results suggest that most EVs are unlikely to be functional for miRNA transfer and raise the question of what EV type is functional for miRNA intercellular communication. However, these numbers would be sufficient for repression in a cell that captured several EVs, and EV miRNA accumulation in the recipient cell is necessary for miRNA function. Since Valadi *et al* [91] reported that miRNAs could be transferred between cells via exosomes, others have described antiviral functions for EVs derived from infected and uninfected cells [20, 32, 92]. Delorme-Axford *et al* [34] demonstrated that cultured primary human placental trophoblasts are highly resistant to infection by some viruses and, importantly, confer this resistance to nonplacental recipient cells by exosome-mediated delivery of specific miRNAs. This study highlights the functional importance of the miRNA content of EVs in an infectious context.

Cellular miRNA content during HIV infection has been suggested as a biomarker of the early/acute pathological stages [93], systemic inflammation [94], and immune activation and disease progression [13]. Many PBMC-focused studies reported cellular miRNA profile changes during HIV infection [35, 43, 54, 93, 94]. We found no difference between PLWH and control participants in terms of miR-92, miR155, and miR-223 levels associated with PBMCs and PMNs although differential regulation of miRNA in PBMCs of HIV-infected, viremic individuals, and control participants have been reported [38]. Correlations between PMN or PBMC miRNA contents and CD4 or CD8 T-cell count suggest that these molecules could provide helpful information for HIV infection management. In conclusion, this study allowed us to improve the technique of EV purification and revealed that a subset of EVs is selectively enriched in miRNA. We also demonstrated that EVs and their miRNA contents might provide clinical markers of physiological status and disease states. Although studies of EV function currently have limitations, our results show that profiling miRNA in plasma EV subsets (rather than focusing on exosomes) and miRNA expression per vesicle could provide a novel and promising approach in the context of HIV immunopathogenesis. Specifically, it could be used to assess immune activation and inflammation in treated HIV patients. As a minimally invasive tool, EV analysis could become very useful for biomedical research and the diagnosis/prognosis of immunological status or comorbidities associated with HIV infection, perhaps even leading to new immunomodulatory therapeutic targets. In the long-term, identification of biomarkers coupled with our increasing knowledge of the pathogenesis will suggest new treatment approaches specifically targeting the associated mechanism.

## SUPPLEMENTARY DATA

Supplementary materials are available at the *Pathogens and Immunity* website. Supplementary data may be provided by the authors to benefit the reader. Supplementary data are not copyedited and are the sole responsibility of the authors. Questions or comments related to supplementary materials should be addressed to the corresponding author.
