# Supplementary material for: Plasma Extracellular Vesicle Subtypes May be Useful as Potential Biomarkers of Immune Activation in People With HIV
Source: Pathog Immun. 2021 Jan 14;6(1):1–28. doi: 10.20411/pai.v6i1.384 (PMC8109236; doi:10.20411/pai.v6i1.384)
Supplement: Supplementary Figure 1 [file pai-6-001-s001.pdf]

A

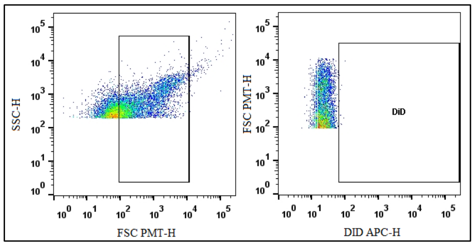
SSC-H
FSC PMT-H
FSC PMT-H
DiD
DID APC-H

B

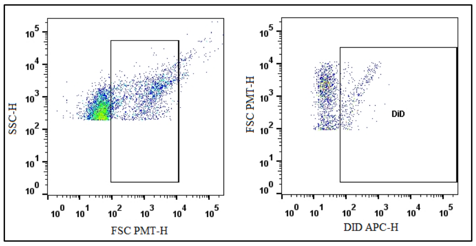
SSC-H
FSC PMT-H
FSC PMT-H
DiD
DID APC-H

C

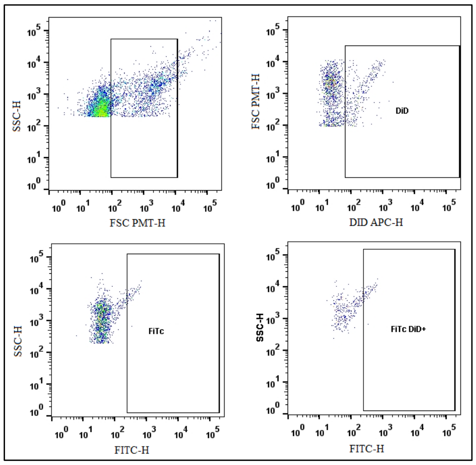
SSC-H
FSC PMT-H
FSC PMT-H
DiD
DID APC-H
SSC-H
FITc
FITC-H
SSC-H
FITc DiD+
FITC-H

D

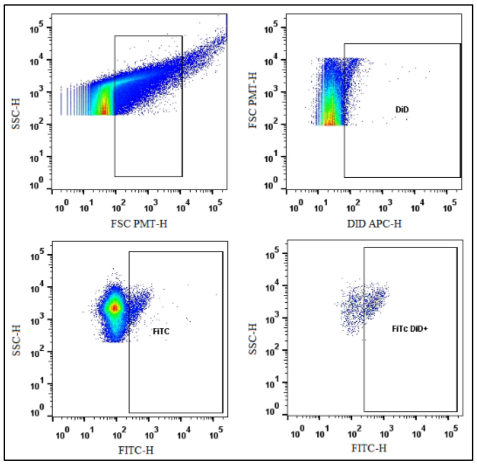
SSC-H
FSC PMT-H
FSC PMT-H
DiD
DID APC-H
SSC-H
FITC
FITC-H
SSC-H
FITc DiD+
FITC-H

E

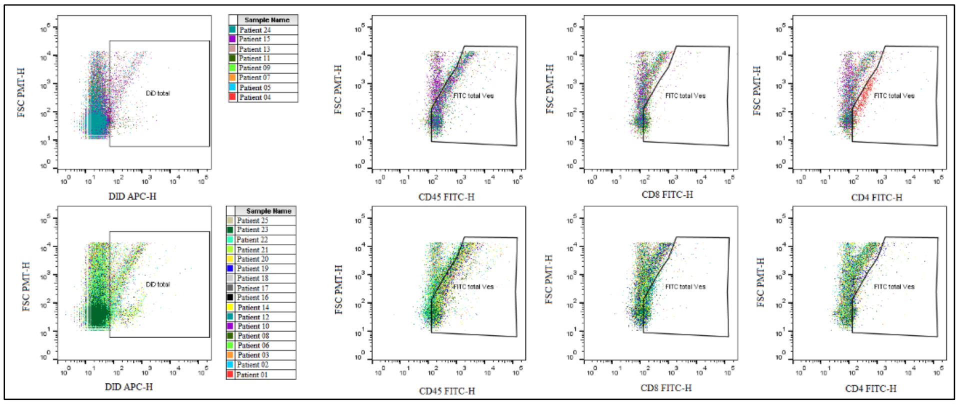
FSC PMT-H
DiD total
DID APC-H
Sample Name
Patient 24
Patient 15
Patient 13
Patient 11
Patient 09
Patient 07
Patient 05
Patient 04
FITC total Ves
CD45 FITC-H
CD8 FITC-H
CD4 FITC-H
Sample Name
Patient 25
Patient 23
Patient 22
Patient 21
Patient 20
Patient 19
Patient 18
Patient 17
Patient 16
Patient 14
Patient 12
Patient 10
Patient 08
Patient 06
Patient 03
Patient 02
Patient 01

F

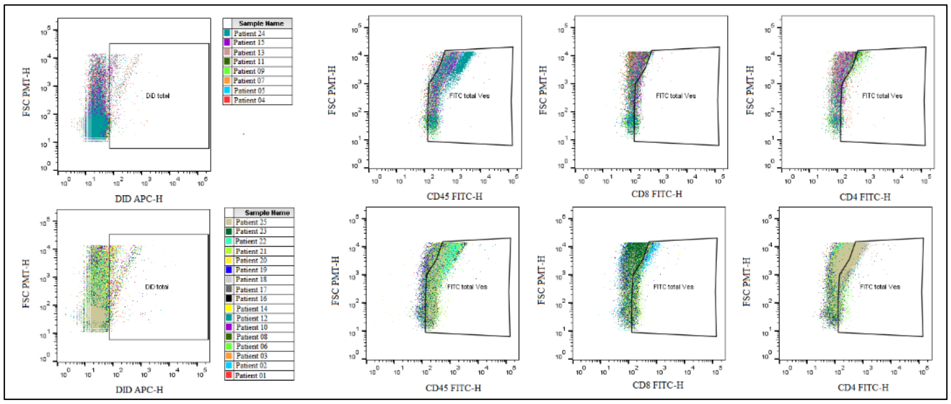
FSC PMT-H
DiD total
DID APC-H
Sample Name
Patient 24
Patient 15
Patient 13
Patient 11
Patient 09
Patient 07
Patient 05
Patient 04
FITC total Ves
CD45 FITC-H
CD8 FITC-H
CD4 FITC-H
Sample Name
Patient 25
Patient 23
Patient 22
Patient 21
Patient 20
Patient 19
Patient 18
Patient 17
Patient 16
Patient 14
Patient 12
Patient 10
Patient 08
Patient 06
Patient 03
Patient 02
Patient 01

Figure S1. Sequential gating to identify specific EV subsets

Large and small EVs purified from HIV-infected (n = 17) and uninfected (n = 8) patients' plasma were incubated with DiD and subsequently labeled with anti-C45, CD8, and CD4-FITC antibodies. A gate, including events from 100 to 1000 nm in diameter based on the beads (FSC-PMT-H) and positive fluorescent events, was used to detect EVs and portrayed in an SSC/FSC-PMT graph. Total DiD+ events are detected in the 100 to 1000 nm gate, and the quantity of FITC+ EVs is determined in the DiD+ EVs gate. A Buffer only (0.22-µmm filtered pore size membrane PBS) was used to determine the background signal. Then each antibody and dye were incubated in PBS in the absence of an EV sample and acquired to determine any particle signal arising from reagents. (**A**) Shows the scatter intensity of buffer (filtered 0.22-µmm pore size membrane PBS) alone, and (**B**) similar results with the addition of the lipophilic dye carbocyanine DiD to buffer only (no EVs). (**C**) Shows an example of a gating strategy with buffer only and with FITC-conjugated antibodies added (no EVs added). (**D**) Shows the gating strategy applied to an EV sample (patient 02). (**E**) Presents a pooling result of large EVs; upper panel shows DiD total EVs, CD45, CD8, and CD4 EVs for control patients (HIV uninfected n = 8) and lower panel for HIV-infected patients (n = 17). (**F**) Presents similar results for small EVs.
