# Supplementary material for: Plasma Extracellular Vesicle Subtypes May be Useful as Potential Biomarkers of Immune Activation in People With HIV
Source: Pathog Immun. 2021 Jan 14;6(1):1–28. doi: 10.20411/pai.v6i1.384 (PMC8109236; doi:10.20411/pai.v6i1.384)
Supplement: Supplementary Figure 2 [file pai-6-001-s002.pdf]

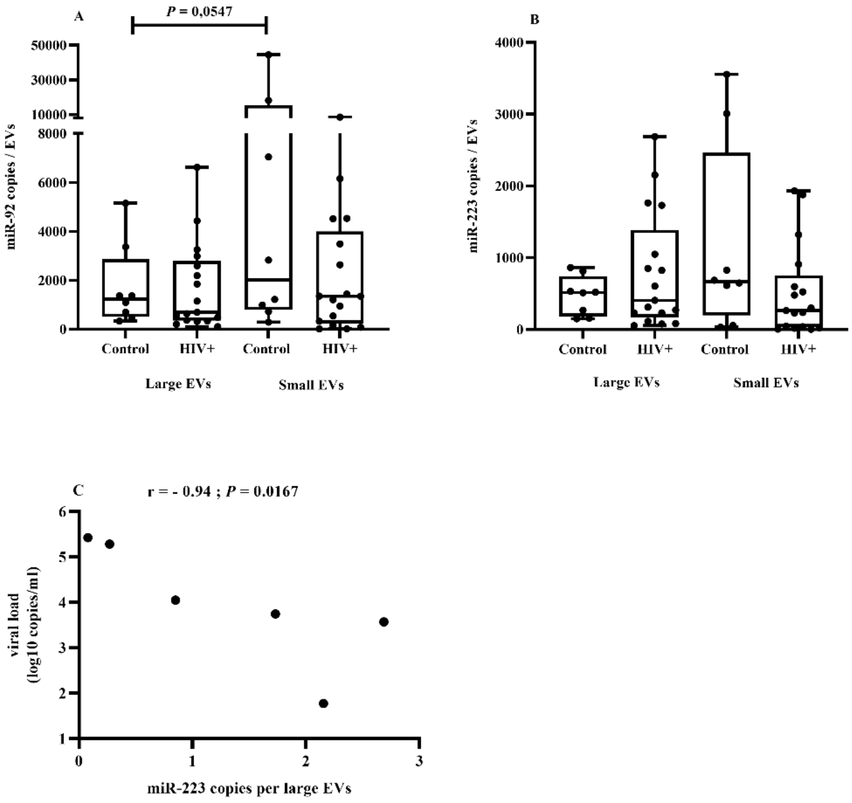

Figure S2: EV miRNA level expression as copies per vesicle and correlation with HIV-relevant clinical parameters

Copy number of each molecule per large and small vesicle in ml of plasma is expressed for each subject (n = 25); (**A**) miR-92 and (**B**) miR-223. Box and whisker plots show the overall ranges and medians. Spearman tests were used to evaluate correlations between viral load and miR-223 copies per large vesicle (**C**) in ART-naive patients.
